# High-Quality Genome Assemblies of 4 Members of the *Podospora anserina* Species Complex

**DOI:** 10.1093/gbe/evae034

**Published:** 2024-02-22

**Authors:** S Lorena Ament-Velásquez, Aaron A Vogan, Ola Wallerman, Fanny E Hartmann, Valérie Gautier, Philippe Silar, Tatiana Giraud, Hanna Johannesson

**Affiliations:** Division of Population Genetics, Department of Zoology, Stockholm University, 106 91 Stockholm, Sweden; Systematic Biology, Department of Organismal Biology, Uppsala University, 752 36 Uppsala, Sweden; Department of Medical Biochemistry and Microbiology, Comparative Genetics and Functional Genomics, Uppsala University, 752 37 Uppsala, Sweden; Ecologie Systematique Evolution, CNRS, Université Paris-Saclay, AgroParisTech, 91198 Gif-sur-Yvette, France; Laboratoire Interdisciplinaire des Energies de Demain (LIED), Université de Paris Cité, F-75013 Paris, France; Laboratoire Interdisciplinaire des Energies de Demain (LIED), Université de Paris Cité, F-75013 Paris, France; Ecologie Systematique Evolution, CNRS, Université Paris-Saclay, AgroParisTech, 91198 Gif-sur-Yvette, France; Systematic Biology, Department of Organismal Biology, Uppsala University, 752 36 Uppsala, Sweden; The Royal Swedish Academy of Sciences, 114 18 Stockholm, Sweden; Department of Ecology, Environmental and Plant Sciences, Stockholm University, 106 91 Stockholm, Sweden

**Keywords:** Sordariales, *Podospora bellae-mahoneyi*, *Podospora pseudoanserina*, *Podospora pseudopauciseta*, *Podospora pseudocomata*, chromosomal rearrangements, phylogenomics

## Abstract

The filamentous fungus *Podospora anserina* is a model organism used extensively in the study of molecular biology, senescence, prion biology, meiotic drive, mating-type chromosome evolution, and plant biomass degradation. It has recently been established that *P. anserina* is a member of a complex of 7 closely related species. In addition to *P. anserina*, high-quality genomic resources are available for 2 of these taxa. Here, we provide chromosome-level annotated assemblies of the 4 remaining species of the complex, as well as a comprehensive data set of annotated assemblies from a total of 28 *Podospora* genomes. We find that all 7 species have genomes of around 35 Mb arranged in 7 chromosomes that are mostly collinear and less than 2% divergent from each other at genic regions. We further attempt to resolve their phylogenetic relationships, finding significant levels of phylogenetic conflict as expected from a rapid and recent diversification.

**Significance**

Here, we provide a data set of 28 annotated genomes from the *Podospora anserina* species complex, including chromosome-level assemblies of 4 species that lacked a reference genome. With this data set in hand, biologists can take advantage of the molecular tools available for *P. anserina* to study evolutionary dynamics at the intersection between micro- and macroevolution, with particular emphasis on trait evolution, genome architecture, and speciation.

## Introduction

The filamentous fungus *Podospora anserina* (order Sordariales) holds significant importance as a model for understanding ascomycete biology and beyond (Silar 2013). It has proved particularly valuable in advancing the study of molecular biology, senescence, heterokaryon incompatibility, sexual reproduction, prion biology, meiotic drive, and plant biomass degradation (Pinan-Lucarré et al. 2007; Silar 2013, 2020; Grognet et al. 2014; Hamann and Osiewacz 2018; Hartmann et al. 2021; Vogan et al. 2022). Its reference genome was published as early as 2008 (Espagne et al. 2008), followed by chromosome-level assemblies of several wild-type strains (Vogan et al. 2021, 2019) and short-read population genomic data from Wageningen, The Netherlands, as well as a few strains from France and other localities (Ament-Velásquez et al. 2022). However, knowledge of its diversity, geographic distribution, ecology, and evolution lags behind. It is generally agreed that *P. anserina* is an obligately sexual coprophilous fungus, but there are observations of potential asexual spores (Boucher et al. 2017; Silar 2020) and endophytic stages (Matasyoh et al. 2011). The name *P. anserina* itself has been riddled with taxonomic uncertainties (Ament-Velásquez et al. 2020; Silar 2020), leading to confusion regarding the exact identity of the fungal material used in some studies. Unsurprisingly, a phylogenetic survey showed that many strains commonly regarded as *P. anserina* actually belong to at least 6 additional species scattered around the world (Boucher et al. 2017). Representatives of all these species have been sequenced with short-read technology, which was useful to explore the dynamics of recombination suppression around the mating-type locus (Hartmann et al. 2021). However, long-read data are necessary to understand the evolution and genetic basis of many traits. For example, the development of high-quality genomic resources of 2 of these species, *Podospora comata* and *Podospora pauciseta*, already provided important insights into the evolutionary dynamics of selfish genetic elements and genome architecture (Silar et al. 2018; Vogan et al. 2021, 2019).

As originally defined, only 1 or 2 strains are known for most members of the *P. anserina* species complex (Boucher et al. 2017), many available at the Westerdijk Fungal Biodiversity Institute Collection (identified with CBS numbers). All species have a similar morphology, mating system, and coprophilous habit, with the exception of the only known strain of *Podospora pseudocomata*, which was isolated from soil (Boucher et al. 2017; Hartmann et al. 2021). Despite their similarities, they are considered biological species, since there is reproductive isolation in the form of low mating success and female sterility in the hybrids (Boucher et al. 2017). Moreover, they are identifiable by differences at the fungal barcode ITS, as well as other nuclear markers (Boucher et al. 2017). Previous genomic comparisons showed that *P. anserina*, *P. comata*, and *P. pauciseta* are more than 98% identical in genic regions (Vogan et al. 2019), confirming that they are very closely related. However, their exact relationships remain unresolved. In this study, we generated chromosome-level annotated genome assemblies of the 4 remaining species (*Podospora bellae-mahoneyi*, *Podospora pseudoanserina*, *Podospora pseudopauciseta*, and *P. pseudocomata*), as well as short-read data from additional strains. In addition, we conducted a phylogenomic analysis to provide an evolutionary framework for addressing the variety of questions for which *Podospora* is well suited.

## Results and Discussion

### Genome Assemblies and Annotation

We isolated haploid cultures (of mating type + or −) from dikaryotic strains. From those, we selected 1 strain of each of the 4 species that lack a reference genome (hereafter, the focal strains) for Oxford Nanopore MinION and Illumina HiSeq sequencing (supplementary table S1, Supplementary Material online). In addition, we sequenced with Illumina HiSeq a known strain of *P. pauciseta* (CBS 451.62+), the type strain of *P. pseudoanserina* (CBS 253.71+), and 2 newly collected *P. comata* strains (Wageningen Collection numbers Wa132+ and Wa133−). Along with previously published assemblies and sequencing data of other members of the species complex, we assembled and annotated a total of 28 *Podospora* genomes, including the type strains of all 7 species (supplementary table S1, Supplementary Material online).

**Fig. 1.**
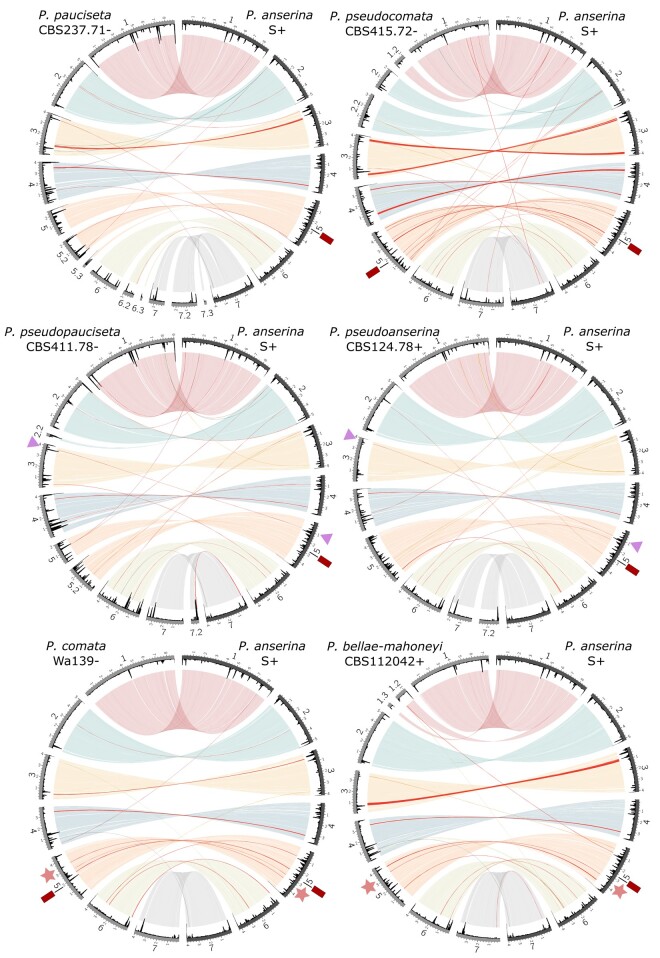
Circos plots comparing the reference assembly of *P. anserina* (strain S+, right side of each plot) to the best genome assembly of each of the other members of the species complex (left side). Light colors correspond to NUCmer alignments (larger than 5 kb) of the different chromosomes as defined in *P. anserina* (chr. 1: red; chr. 2: turquoise; chr. 3: yellow; chr. 4: blue; chr. 5: orange; chr. 6: olive green; chr 7: gray). Red solid links mark chromosome inversions or inverted translocations. The internal track in black is a histogram of repetitive element abundance calculated in sliding windows of 50 kb with steps of 10 kb. The stars and triangles mark shared structural variants (relative to *P. anserina*). The location of the insertion in chr. 5 discussed in the text is marked with a red square.

Whole genome assemblies of Oxford Nanopore MinION data from the focal strains recovered mostly chromosome-level scaffolds that are highly collinear with the reference genome of *P. anserina* (Fig. 1), although *P. pseudocomata* (strain CBS415.72−) has slightly more rearrangements. Thus, all species in the complex likely have 7 chromosomes, a similar genome size of around 35 Mb, and a repeat content ranging from more than 3% (*P. comata*) to around 7% (*P. pseudopauciseta*; supplementary table S1, Supplementary Material online) that is mostly concentrated in clusters (Fig. 1). Genome annotation using previously published RNA-seq data of *P. anserina* and *P. comata* (Vogan et al. 2021; Lelandais et al. 2022) resulted in similar protein-coding gene numbers for assemblies produced with long-read data, from 11,033 (*P. bellae-mahoneyi* strain CBS112042+) to 11,727 (*P. anserina* strain T_G_+) genes, while the *P. anserina* reference itself has 10,803 predicted protein-coding genes (supplementary table S1, Supplementary Material online). The discrepancy in gene numbers with the reference is likely due to differences in the annotation method (with our pipeline, we recovered 11,660 genes in the *P. anserina* reference assembly). Similarly, the annotation of long-read assemblies gave comparable BUSCO numbers to the reference genome of *P. anserina* (supplementary table S1, Supplementary Material online), specifically 96.6% to 98.2% conserved proteins present. The annotation of short-read assemblies resulted in 90% to 93.5% conserved proteins, although assemblies alone reached BUSCO values more in line with the long-read assemblies (supplementary table S1, Supplementary Material online).

### Phylogenomics and Comparative Genomics

**Fig. 2.**
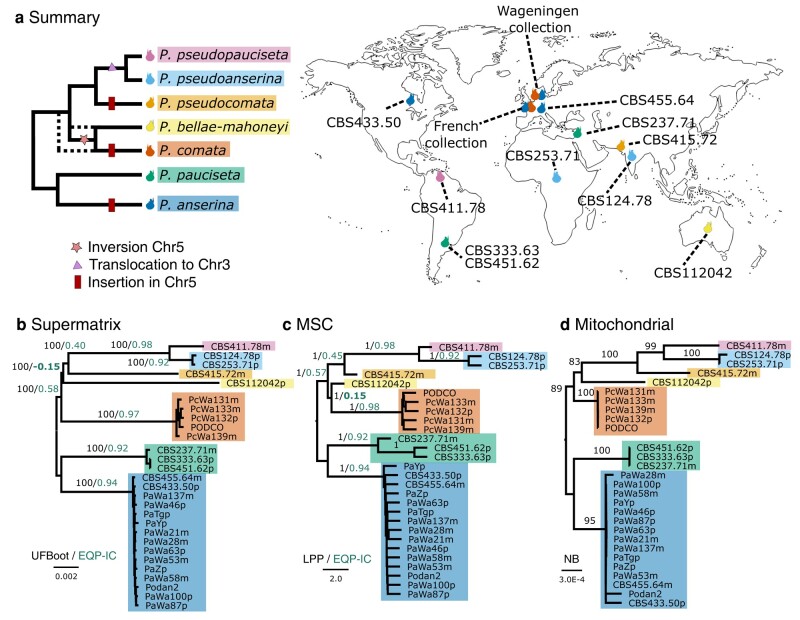
Phylogenetic relationships of the *Podospora* strains with genomic resources and their geographic distribution. a) Summary cladogram based on the phylogenomic analyses and the detected structural variants, with dotted branches illustrating an alternative topology. Fruiting body cartoons mark the country where the different strains were sampled. Phylogenetic relationships were inferred from a supermatrix ML analysis b) or a MSC analysis c) of nuclear genes, as well as a ML analysis of concatenated mitochondrial genes d). Rooting is tentative based on analyses with *C. samala* as an outgroup. Branch lengths of the phylograms are drawn to scale as indicated by the scale bar (nucleotide substitutions per site in b) and d) and coalescent units in c)). Different support metrics are shown next to their corresponding branches (within-species values are removed for clarity). The EQP-IC value of the conflicting branch is highlighted in bold. MSC, multispecies coalescent; UFBoot, ultrafast bootstrap; EQP-IC, extended quadripartition internode certainty; LPP, local posterior probability; NB, nonparametric bootstrap.

We aimed at providing a phylogenetic context of the *P. anserina* species complex by using the 28 genome assemblies (Fig. 2; supplementary table S1, Supplementary Material online). The closest known relative of the *P. anserina* species complex is *Cercophora samala*, strain CBS 307.81 (Ament-Velásquez et al. 2020). Preliminary phylogenomic analyses using CBS 307.81 as the outgroup placed the clade of *P. anserina* and *P. pauciseta* as sister to the other *Podospora* species (supplementary fig. S1, Supplementary Material online). However, this *C. samala* strain is in fact too divergent (around 86% identity in nuclear genic regions to any *Podospora* species) relative to the species complex (>98% identical to each other), potentially creating long-branch attraction (Felsenstein 1981; Emms and Kelly 2017). Hence, only *Podospora* strains were considered below, and we tentatively rooted the phylogeny using *P. anserina* and *P. pauciseta*.

A summary of our results is found in Fig. 2a. We inferred groups of single-copy orthologous (SCO) genes and used them to produce 3 different phylogenetic analyses (Fig. 2b to d). All phylogenies resulted in well-supported species-level clades separated by very short internal branches, suggesting rapid diversification. Most relationships were congruent among analyses, except with regard to the relative positions of *P. comata* and *P. bellae-mahoneyi*. In the first analysis, a maximum likelihood (ML) phylogeny produced from a supermatrix of 1,000 nuclear SCO genes, *P. bellae-mahoneyi* was inferred as sister to the clade containing *P. pseudopauciseta*, *P. pseudoanserina*, and *P. pseudocomata* (Fig. 2b). In contrast, a multispecies coalescent (MSC) analysis of all the 8,596 SCO genes recovered *P. comata* and *P. bellae-mahoneyi* as sister taxa (Fig. 2c). Lastly, a ML phylogeny of 8 mitochondrial genes was in support of the nuclear supermatrix tree, albeit with modest bootstrap values (Fig. 2d). To further explore this phylogenetic conflict, we obtained extended quadripartition internode certainty (EQP-IC) values (Zhou et al. 2020) for the 2 competing topologies. The EQP-IC score can range from 1 to −1. A score of 1 implies that all the SCO gene trees agree with a given branch, while a value of −1 reveals that all trees support an alternative topology. The score approaches 0 if the alternative topologies have similar frequencies among the gene trees (Zhou et al. 2020). As expected, we found intermediate positive values in most internal branches, which might be explained by incomplete lineage sorting (ILS) or introgression. The contesting branches in particular obtained values very close to 0, but there is slight support for the existence of a clade containing *P. comata* and *P. bellae-mahoneyi* (Fig. 2b and c).

Taking advantage of the extensive collinearity between genomes, we also attempted to find phylogenetically informative structural variants. In support of the MSC analysis, we found a medium-scale (33.89 kb, 13 genes) inversion in chromosome 5, relative to *P. anserina*'s genome, that is shared between *P. comata* and P. *bellae-mahoneyi* (Figs. 1 and 2a). In addition, we detected a shared translocation from chromosome 5 to chromosome 3 between *P. pseudoanserina* and *P. pseudopauciseta*, supporting their close sister relationship already observed in the phylogenetic analyses (Figs. 1 and 2a). However, in contradiction with our phylogenies, we found a region in chromosome 5 that is only present in *P. anserina*, *P. comata*, and *P. pseudocomata* (Figs. 1 and 2a). This region ranges from over 40 kb in *P. anserina* to just over 6 kb in *P. pseudocomata* and contains a number of genes and different transposable elements (TEs). Upon closer inspection, we found that the edges of this region are flanked by directed repeats of 4 bp, suggesting that this region represents a TE-mediated insertion, resembling the behavior of large TEs found in Pezizomycetes, including *Podospora* (Vogan et al. 2021; Gluck-Thaler et al. 2022). Its phylogenetic distribution might also be a consequence of ILS or introgression.

## Conclusion

Here, we present high-quality annotated genome assemblies of 4 members of the *P. anserina* species complex. Together with the already available genomic resources, these new data build an evolutionary framework for further in-depth studies of this group of fungi. We provide a general idea of the genomic architecture and relationships between the sampled *Podospora* lineages, while illustrating the high levels of phylogenetic conflict that are typical of rapid species radiations. Moreover, we make available a comprehensive genomic data set of 28 strains for the study of fungal biology and evolution at shallow divergence scales. Combined with the wealth of molecular biology tools available for the model species *P. anserina*, this data set can be used to explore the evolution and function of pangenome content including metabolic clusters, selfish genetic elements like meiotic drivers and TEs, and the buildup of reproductive barriers in filamentous fungi.

## Materials and Methods

All bioinformatic pipelines were done in Snakemake v. 7.25.0 or v. 7.32.3 (Mölder et al. 2021) and are available at https://github.com/SLAment/PodosporaGenomes, unless otherwise stated. Some of these pipelines rely on the Environment for Tree Exploration (ETE3) toolkit v. 3.1.3 (Huerta-Cepas et al. 2016).

### Fungal Material

For detailed information about the strains used, see supplementary table S1 and Supplementary Material, Supplementary Material online. The strains with code starting with “CBS” were originally obtained from the CBS-KNAW Collection (https://wi.knaw.nl/Collection). In addition, we isolated 2 new strains of *P. comata* from rabbit dung collected in the area between Wageningen and Arnhem, The Netherlands (51°58′41.8″N, 5°50′39.6″E, locality Unksepad Oosterbeek) in September of 2016, which were deposited in the Wageningen Collection at the Laboratory of Genetics of the Wageningen University and Research (codes Wa132 and Wa133).

### DNA Extraction and Sequencing

For Illumina sequencing, we grew the haploid strains on Petri dishes of either M2 medium (CBS124.78+ and CBS307.81−) or PASM0.2 (other strains) for 3 to 4 d (Vogan et al. 2019; Silar 2020). We scraped mycelium off the plates in order to obtain about 80 to 200 mg of mycelium per strain and stored it in 1.5-mL Eppendorf tube at −80 °C for at least 24 h before extraction. For most strains, whole genome DNA was extracted with the Fungal/Bacterial Microprep kit (Zymo; https://zymoresearch.eu/). In the case of CBS124.78+ and CBS307.81−, the mycelium was lyophilized for about 20 h, and DNA was extracted using the commercial Nucleospin Soil kit from Macherey Nagel. Paired-end libraries (150 bp reads) were sequenced by either the SNP and SEQ Technology platform (SciLifeLab, Uppsala, Sweden) on the Illumina HiSeq X platform (most strains) or by the high-throughput sequencing core facility of I2BC, Université Paris-Saclay (Centre de Recherche de Gif—http://www.i2bc.paris-saclay.fr/) on the Illumina NextSeq500 platform (CBS124.78+ and CBS307.81−).

For MinION Oxford Nanopore sequencing, we grew the strains in liquid cultures of 3% malt extract solution as in Vogan et al. (2019). High-molecular-weight DNA was extracted as in Sun et al. (2017), using the Genomic Tip G-500 columns (Qiagen) and the PowerClean DNA Clean-Up kit (MoBio Labs). The strain CBS 411.78− was prepared and sequenced with the ligation kit SQK108 in a 1-pot reaction using 500 ng DNA for end-prep and ligation (NEB Ultra-II ligase) and sequenced on an R9.4.1 flow cell. In addition, a rapid barcoding (RBK004) was made for CBS 411.78− to get sufficient coverage for assembly. Similarly, CBS 415.72− was also sequenced using both the RBK004 and LSK108 kit on an R9.4.1 flow cell to maximize yield. CBS 112042+ and CBS 124.78+ were sequenced on R9.4.1 flow cells using the LSK108 kit with 3 μg DNA as input for the end-prep reaction (NEB Ultra-II EP, 20 min at 20 °C and 20 min at 65 °C). Bead purification (SpeedBeads, GE) was done before ligation to deplete short fragments, and 1.5 μg DNA per sample was ligated to 20 μL AMX 1D using Blunt/TA ligase (30 min).

### Genome Assembly

The paired-end HiSeq Illumina reads were cleaned from adapters using cutadapt v. 1.13 (Martin 2011) and Trimmomatic v. 0.36 (Bolger et al. 2014) with the following options: ILLUMINACLIP:adapters.fasta:1:30:9 LEADING:20 TRAILING:20 SLIDINGWINDOW:4:20 MINLEN:30, as in Vogan et al. (2019). We used both forward and reverse paired-end reads in downstream analyses. De novo assemblies were produced as in Vogan et al. (2019). Specifically, we assembled the MinION reads with mean Phred quality (QV) above 9 and longer than 1 kb using Minimap2 v. 2.11 and Miniasm v. 0.2 (Li 2018, 2016). Racon v. 1.3.1 (Vaser et al. 2017) was used twice to polish the resulting assemblies based on the unfiltered reads. We further polished 5 times using the Illumina reads with Pilon v. 1.22 (Walker et al. 2014), which were mapped using BWA v. 0.7.17 (Li and Durbin 2009), with PCR duplicates marked by Picard v. 2.18.11 (http://broadinstitute.github.io/picard/) and with local indel realignment from the Genome Analysis Toolkit (GATK) v. 3.7 (Van der Auwera et al. 2018). For the strains without long-read data, we ran SPAdes v. 3.12.0 (Bankevich et al. 2012) with the *k*-mers 21,33,55,77 (most strains) or 21,29,37,45,53,61,79,87 (CBS307.81−) and the --*careful* option. Due to high numbers of read pairs with read mates mapping on different chromosomes during the read mapping procedure (about 20% of mapping read pairs), we used each paired-end sequencing run as 2 independent single-end sequencing runs for read mapping and de novo assembly of CBS124.78+ and CBS307.81−, respectively (see Hartmann et al. 2021).

In the case of samples with long-read data, the scaffolds were assigned to chromosomes and reoriented by mapping them to the reference genome of the strain S (Espagne et al. 2008), which is available at the Joint Genome Institute MycoCosm website (https://mycocosm.jgi.doe.gov/Podan2/Podan2.home.html) as “Podan2”. Mapping was performed with the NUCmer program from the MUMmer package v. 4.0.0beta2 (Kurtz et al. 2004) using the parameters *-b 2000 -c 200 --maxmatch*. Contigs smaller than 100 kb that contained rDNA repeats or mitochondrial sequences were discarded (except for the largest mitochondrial contig). Genome quality statistics were calculated with QUAST v. 4.6.3 (Mikheenko et al. 2016). Mean depth of coverage was obtained using Qualimap v.2.2 (Okonechnikov et al. 2016). We also used BUSCO v. 5.3.1 (Manni et al. 2021) with the 3,817 Sordariomycetes_odb10 ortholog set to assess assembly completeness. As dependencies, we used BLAST suit 2.12.0+ (Camacho et al. 2009), AUGUSTUS v. 3.4.0 (Stanke and Waack 2003), and HMMER v. 3.2.1 (Mistry et al. 2013).

We verified the correct assembly of the mitochondrial contig in the focal strains CBS 112042+, CBS 124.78+, CBS 411.78−, and CBS 415.72−, by visual inspection of long- and short-read mapping. We found a misassembly in the contig of CBS 415.72− around the first exon of the *cox1* gene. Hence, we extracted the long reads mapped to this mitochondrial contig using the *bam2fq* option of SAMtools v. 1.17 (Danecek et al. 2021) and reassembled them with Flye v. 2.9.1 (Kolmogorov et al. 2020) with the arguments --*iterations 2 --meta --keep-haplotypes*. We recovered 2 circular contigs, 1 of which proved to be formed by multiple tandem repeats of the first part of *cox1*, a configuration known as α senDNA or plDNA (Cummings et al. 1985; Hamann and Osiewacz 2018). The other contig corresponded to the full mitochondrion. We polished the mitochondrial contig 3 times using the Illumina reads as above and discarded the plDNA. We manually recircularized the mitochondrial contigs of the focal strains to avoid breaking genes.

### Genome Annotation

The annotation of all genomes was done with a modified version of a previous pipeline (Vogan et al. 2021). Briefly, we used previously produced (Vogan et al. 2019) training files for SNAP release 2013-11-29 (Lomsadze et al. 2005) and GeneMark-ES v. 4.38 (Lomsadze et al. 2005; Ter-Hovhannisyan et al. 2008) within the program MAKER v. 3.01.04 (Holt and Yandell 2011; Campbell et al. 2014) to generate gene models for all species. MAKER was run with the following dependencies: BLAST suit 2.13.0+, tRNAscan-SE v. 1.3.1 (Lowe and Eddy 1997), Exonerate v. 2.4.0 (Slater and Birney 2005), and RepeatMasker v. 4.1.0 (http://www.repeatmasker.org/). As external protein evidence, we used the sequences from the PODANS_v2016 annotation (Lelandais et al. 2022) and from the reference genome of *P. comata* (PODCO; Silar et al. 2018), along with a small set of manually curated proteins (available in the GitHub repository). We also used transcript models as external evidence from 2 sources: the curated set of transcripts with defined transcription starts and ends from PODANS_v2016 and transcript models of published RNA-seq data of *P. anserina* and *P. comata* (Vogan et al. 2021, 2019). The latter were produced as in Vogan et al. (2021), using STAR v. 2.7.10b (Dobin et al. 2013), Cufflinks v. 2.2.1 (Trapnell et al. 2010), and TransDecoder v. 5.7.0 (Haas et al. 2013). Additionally, we used the custom library “PodoTE-1.00” (https://github.com/johannessonlab/SpokBlockPaper/blob/master/Annotation/data/) to annotate repeated elements in all species using RepeatModeler v. 1.0.8 (http://www.repeatmasker.org/RepeatModeler/).

The gene models produced by the MAKER pipeline were functionally annotated with Funannotate v. 1.8.15 (Palmer and Stajich 2020) using the *annotate* function with the dependencies HMMER 3.3.2 (Mistry et al. 2013), Diamond v. 2.1.6 (Buchfink et al. 2021) with the UniProt DB version 2023_01, InterProScan v. 5.62-94.0 (Jones et al. 2014; Blum et al. 2021), bedtools v. 2.30.0 (Quinlan and Hall 2010), Eggnog-mapper v. 2.1.10 (Cantalapiedra et al. 2021) with the database emapperdb-5.0.2, and the fungal version of antiSMASH v. 6.1.1 (Blin et al. 2021). The input assemblies were soft masked with RepeatModeler as above. Mitochondrial annotation was done with the online version of MFannot (Lang et al. 2023), setting the genetic code to 4 (https://megasun.bch.umontreal.ca/apps/mfannot/; consulted in July 2023; see Supplementary material for details.

### Comparative Genomics

In order to explore collinearity among the *Podospora* species, we used NUCmer with parameters *-b 2000 -c 2000 --maxmatch* to align the long-read assembly of a representative of each species against Podan2 (Fig. 1). We further calculated the coverage distribution of repetitive elements along chromosomes using the RepeatMasker annotation and by dividing the genome in windows of 50 kb with steps of 10 kb using the utilities *makewindows* and *coverage* of BEDTools v. 2.29.0 (Quinlan and Hall 2010; Quinlan 2014). Both the alignments and coverage distributions were plotted using Circos v. 0.69.6 (Krzywinski et al. 2009). We removed all alignments smaller than 5 kb to exclude the most abundant TEs.

### Phylogenomic Analyses

In order to resolve the relationships between the *Podospora* species, we inferred SCO groups by running OrthoFinder v. 2.5.2 (Emms and Kelly 2019) with a single representative per species: strains S+ (Podan2), PODCO, CBS 237.71−, CBS 124.78+, CBS 411.78−, CBS 415.72, and CBS 112042+. OrthoFinder was run with the proteins predicted for the chosen strains. However, the low level of divergence within the species complex makes the proteins largely uninformative. Hence, once SCO groups were defined, we used the ortholog of the reference S+ as a BLASTn query to retrieve the nucleotide sequences of the corresponding homologs (including introns) in all the *Podospora* strains in our data set (supplementary table S1, Supplementary Material online). We kept only the orthogroups with a single sequence per strain, resulting in a total of 8,596 SCO groups. These were then aligned with MAFFT v. 7.407 (Katoh and Toh 2008) with the options --*adjustdirection --anysymbol --maxiterate 1000 --retree 1 --localpair*. We inferred ML trees of each alignment using IQ-TREE v. 2.2.3 (Nguyen et al. 2015; Hoang et al. 2017) with parameters *-m MFP -seed 1234 -bnni --keep-ident -bb 1000*. To reduce noise in our data set, we collapsed branches with ultrafast bootstraps (UFBoots) support lower than 95% into polytomies using Newick utilities v. 1.6 (Junier and Zdobnov 2010). These trees were then given to ASTRAL v. 5.7.3 (Zhang et al. 2018) to construct a MSC phylogeny. In addition, we randomly selected 1,000 of the SCO to form a concatenated alignment (supermatrix) of 1,732,364 sites (39,771 [2.4%] informative) and produced a ML phylogeny with IQ-TREE as above. In order to evaluate the level of conflict within the SCO trees with respect to both the supermatrix ML and MSC phylogenies, we calculated the EQP-IC score with the program QuartetScores v. 1.0 (Zhou et al. 2020) using the unrooted reference trees and the SCO trees with collapsed low-support branches from above as the evaluation set (see Supplementary material for details).

## Supplementary Material

evae034_Supplementary_Data

## Data Availability

The genome assemblies and raw sequencing data are available at GenBank under the BioProject PRJNA685103 (see supplementary table S1, Supplementary Material online). The reads of the strains sequenced with long-read technology in this study have BioSample accessions SAMN17076437 (CBS124.78+), SAMN17076438 (CBS411.78−), SAMN17076439 (CBS415.72−), and SAMN17076440 (CBS112042+). The annotated genome assemblies are available in GenBank under accession numbers JAFFHC000000000, JAFFHB000000000, JAFFHA000000000, and JAFFGZ000000000. The strains newly sequenced with Illumina have BioSample accessions SAMN37845441 (Wa132+), SAMN37845442 (Wa133−), SAMN37845443 (CBS 451.62+), and SAMN37845444 (CBS 253.71+). In addition, all genome assemblies, annotations in gff3 format, and alignments are available in Dryad Digital Repository (https://doi.org/10.5061/dryad.1vhhmgr0j). Scripts and Snakemake pipelines are available at https://github.com/SLAment/PodosporaGenomes.
