# Supplementary material for: High-Quality Genome Assemblies of 4 Members of the Podospora anserina Species Complex
Source: Genome Biol Evol. 2024 Feb 22;16(3):evae034. doi: 10.1093/gbe/evae034 (PMC10936905; doi:10.1093/gbe/evae034)
Supplement: evae034_Supplementary_Data [file evae034_supplementary_data.zip › Supplementary_Materials_R1.pdf]

Supplementary Methods of

High-quality genome assemblies of four members of the *Podospora anserina* species complex

S. Lorena Ament-Velásquez, Aaron A. Vogan, Ola Wallerman, Fanny Hartmann, Valérie Gautier, Philippe Silar, Tatiana Giraud, Hanna Johannesson

Notes on fungal material

The newly isolated *P. comata* strains were obtained by incubating the rabbit dung pieces on 9 cm Petri dishes at room temperature until *Podospora*-like perithecia developed (around 10 days). The perithecia were identified based on gross morphology, and moved from the dung into a water agar plate covered with an NC 45 membrane filter (Schleicher & Schuell, Dassel, Germany) and pierced with a sterilized needle to retrieve an ascus with 4 spores. The four spores were isolated into germination media (PASM2 with 5g/L ammonium acetate; Vogan et al., 2019). Two days after germination, the mycelium was stored by inoculating PASM0.2 plates (Vogan et al., 2019). Only one spore was used as representative culture, but all siblings were stored in the Wageningen Collection. For all *Podospora* strains, we obtained haploid cultures by letting them undergo selfing and retrieving sexual ascospores with a single haploid nucleus (i.e., a monokaryotic spore). We indicate the mating type of the monokaryotic cultures with either a + or a - next to the strain number. The sequenced *C. samala* strain was a monokaryotic F1 progeny from strain CBS307.81 (of - mating type, hence referred to as CBS307.81-) (see Hartmann et al., 2021). Notice that a total of 106 *P. anserina* strains from Wageningen were sequenced previously (Ament-Velásquez et al., 2022), but only those with long-read data were selected as representatives for our analyses. All strains from outside of Wageningen or from the other species with available sequence data were also included (**Supplementary Table 1**).

Notes on genome annotation

Manual curation of the output of RepeatModeler v. 1.0.8 (http://www.repeatmasker.org/RepeatModeler/) from long-read assemblies, as produced by the pipelines *PaTEs.smk* and *TEManualCuration.smk* (https://github.com/johannessonlab/SpokBlockPaper), revealed no new TEs in the *P. anserina* species complex except for two elements in *P. pseudocomata*: a novel LINE in that we named *kermit* (no other LINE elements have been identified in the other *Podospora* species), and a new *Ty3* element related to *Yeti* (Hamann et al., 2000) that we called *sasquatch*. In addition, we found that the previously unclassified element *leptodactylodon* is an LTR element and that *P. pseudocomata* has a full copy of the *rana* LTR, which was previously only known from solo elements (Espagne et al., 2008). These new sequences are part of PodoTE-1.00 library. The total repeat content in a genome was estimated from the output of RepeatMasker as the percentage of sites annotated as repeats out of the total in the assembly (excluding mitochondrial contigs in long-read assemblies) with the script “totalcovergff.py” v. 2.2 available at https://github.com/SLAment/Genomics/tree/master/GenomeAnnotation.

The original gene names of the reference S+ strain genome (Espagne et al., 2008; Lelandais et al., 2022) are extensively used by the *Podospora* scientific community and follow a convention that marks the chromosome name and the gene number within the chromosome. For example, “Pa_1_3060” is the gene 3060 in chromosome 1. Hence, we took advantage of the high collinearity between the *Podospora* genomes to identify one-to-one orthologs using BLASTn and name them accordingly. If a focal gene had a single, high-quality hit in the PODANS_v2016 annotation (Lelandais et al., 2022), and this hit was itself the only (high quality) hit of the focal gene, these were considered one-to-one orthologs. A high-quality hit had maximum e-value of 0.001 and an identity equal or higher than 98% (for *P. anserina* strains), 93% (*P. comata* or *P. pauciseta*), 90% (the other *Podospora* species) or 70% (*C. samala*). This simple strategy assigned more than 90% of genes to one-to-one orthologs in all strains. The IDs of these genes were then set to reflect their relationships to the reference genome. For example, the one-to-one ortholog of “Pa_1_3060” was named “QC762_103060” in the strain CBS415.72-, where QC762 is the strain’s NCBI locus tag.

The mitochondrial annotation in tbl format produced by the MFannot program (Lang et al., 2023) was modified with the script *MFannot4ncbi.py* to make it closer to NCBI requirements, and further transformed to a gff3 file with the script *tbl2gff.py* (available at https://github.com/SLAment/Genomics/blob/master/GenomeAnnotation). We performed manual curation of all the canonical protein coding genes, as well as the ribosomal large and small subunit genes, to match the *P. anserina* reference (Genbank accession number NC_001329.3). Considering the high Nanopore error rate and the poor mapping of Illumina reads to mitochondrial contigs during polishing of CBS 112042+ and CBS 411.78- (due to the presence of multiple mitochondrial contigs), we verified that the genes sequence matched the assembly of short reads and corrected them when necessary. The final gff3 file was transformed back to tbl format with *gff3TOtbl.py* (also in the repository above) for submission to NCBI.

Notes on comparative genomics

All inversions and translocations detected in the NUCmer output were manually inspected to identify homologous events between species and marked in the final Circos figure. The shared inversion in *P. comata* and *P. bellae-mahoneyi* compared to the other species is located in the Podan2 genome at coordinates 3440773-3474671 in chromosome 5. We verified that the edges are identical. Similarly, the shared translocation from chromosome 5 in the other species to chromosome 3 in *P. pseudoanserina* and *P. pseudopauciseta* is located at Podan2 coordinates 966739-973648. The insertion shared by *P. anserina*, *P. comata* and *P. pseudocomata* is located at coordinates 2752403-2784937 in chromosome 5 of Podan2.

Notes on phylogenomic analyses

To complement the nuclear data, we extracted the coding sequence of seven mitochondrial genes with one or no introns (*atp6*, *cox2*, *cox3*, *nad2*, *nad3*, *nad4*, and *nad6*), as well as the small subunit ribosomal RNA gene (*rns*), from both the long- and short-read assemblies. As the polishing often failed for mitochondrial contigs, we gave priority to the short-read assembly

sequences when discrepancies occurred. We excluded all introns since these are known to be polymorphic in at least *P. anserina* (Belcour et al., 1997). Moreover, the presence of introns and other regions of the mitochondrial genome can depend on the age of the mycelium (Cummings et al., 1985; Hamann and Osiewacz, 2018), and preliminary analyses with multiple-intron genes revealed that polymorphic sites are often correlated with intron presence/type rather than phylogenetic signal, in particular at the edges of exons. The final concatenated alignment of the eight genes contained 8655 sites, including 34 informative sites. We produced ML trees with IQ-TREE as in the main text.

OrthoFinder can estimate the root in a set of species using patterns of gene duplications in the input proteomes (Emms and Kelly, 2017). In our analysis, this method put the root between a clade containing *P. anserina* (S+) and *P. comata* (T_D+) and a sister clade containing all the other species. This result is at odds with all of our other analyses, where *P. anserina* and *P. pauciseta* are sister species. We found this result was driven by the shared gene content in the chromosome 5 insertion, as seven annotated genes within this region are exclusive to those two species and absent in the homologous region of *P. pseudocomata*. Additional manual attempts to find shared gene duplications across the complex failed. Hence, we did not consider this rooting method further.

References

Ament-Velásquez, S.L., Vogan, A.A., Granger-Farbos, A., Bastiaans, E., Martinossi-Allibert, I., Saupe, S.J., de Groot, S., Lascoux, M., Debets, A.J.M., Clavé, C., Johannesson, H., 2022. Allorecognition genes drive reproductive isolation in *Podospora anserina. Nat Ecol Evol* 6, 910–923. https://doi.org/10.1038/s41559-022-01734-x

Belcour, L., Rossignol, M., Koll, F., Sellem, C.H., Oldani, C., 1997. Plasticity of the mitochondrial genome in *Podospora*. Polymorphism for 15 optional sequences: Group-I, group-II introns, intronic ORFs and an intergenic region. *Current Genetics* 31, 308–317. https://doi.org/10.1007/s002940050210

Cummings, D.J., MacNeil, I.A., Domenico, J., Matsuura, E.T., 1985. Excision-amplification of mitochondrial DNA during senescence in *Podospora anserina*. DNA sequence analysis of three unique "plasmids." *Journal of Molecular Biology* 185, 659–680.

Emms, D.M., Kelly, S., 2017. STRIDE: Species Tree Root Inference from Gene Duplication Events. *Molecular Biology and Evolution* 34, 3267–3278. https://doi.org/10.1093/molbev/msx259

Espagne, E., Lespinet, O., Malagnac, F., Da Silva, C., Jaillon, O., Porcel, B.M., Couloux, A., Aury, J.-M., Ségurens, B., Poulain, J., Anthouard, V., Grossetete, S., Khalili, H., Coppin, E., Déquard-Chablat, M., Picard, M., Contamine, V., Arnaise, S., Bourdais, A., Berteaux-Lecellier, V., Gautheret, D., de Vries, R.P., Battaglia, E., Coutinho, P.M., Danchin, E.G., Henrissat, B., Khoury, R.E., Sainsard-Chanet, A., Boivin, A., Pinan-Lucarré, B., Sellem, C.H., Debuchy, R., Wincker, P., Weissenbach, J., Silar, P., 2008. The genome sequence of the model ascomycete fungus *Podospora anserina. Genome Biology* 9, R77. https://doi.org/10.1186/gb-2008-9-5-r77

Hamann, A., Feller, F., Osiewacz, H.D., 2000. Yeti – a degenerate gypsy-like LTR retrotransposon in the filamentous ascomycete *Podospora anserina. Current genetics* 38, 132–40. https://doi.org/10.1007/s002940000144

Hamann, A., Osiewacz, H.D., 2018. *Podospora anserina*: a filamentous fungus with a strong mitochondrial etiology of aging, in: *Conn's Handbook of Models for Human Aging*. Elsevier, pp. 431–444. https://doi.org/10.1016/B978-0-12-811353-0.00031-2

Hartmann, F.E., Ament-Velásquez, S.L., Vogan, A.A., Gautier, V., Le Prieur, S., Berramdane, M., Snirc, A., Johannesson, H., Grognet, P., Malagnac, F., Silar, P., Giraud, T., 2021. Size Variation of the Nonrecombining Region on the Mating-Type Chromosomes in the Fungal *Podospora anserina* Species Complex. *Molecular Biology and Evolution.* https://doi.org/10.1093/molbev/msab040
Lang, B.F., Beck, N., Prince, S., Sarrasin, M., Rioux, P., Burger, G., 2023. Mitochondrial genome annotation with MFannot: a critical analysis of gene identification and gene model prediction. *Front. Plant Sci.* 14, 1222186. https://doi.org/10.3389/fpls.2023.1222186
Lelandais, G., Remy, D., Malagnac, F., Grognet, P., 2022. New insights into genome annotation in Podospora anserina through re-exploiting multiple RNA-seq data. *BMC Genomics* 23, 859. https://doi.org/10.1186/s12864-022-09085-4
Vogan, A.A., Ament-Velásquez, S.L., Granger-Farbos, A., Svedberg, J., Bastiaans, E., Debets, A.J., Coustou, V., Yvanne, H., Clavé, C., Saupe, S.J., Johannesson, H., 2019. Combinations of Spok genes create multiple meiotic drivers in *Podospora*. eLife 8, e46454. https://doi.org/10.7554/eLife.46454

Supplementary Figures

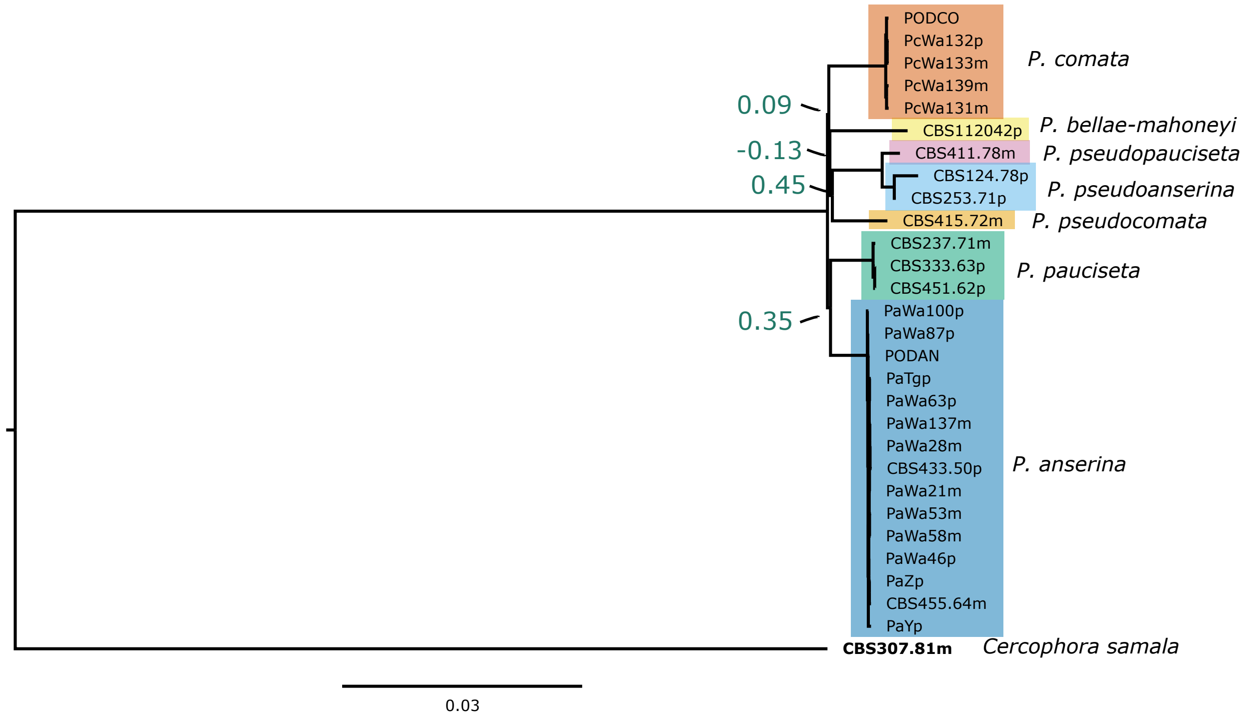

Supplementary Figure 1. Maximum-likelihood analysis of 1000 concatenated nuclear genes. Branch lengths are drawn to scale as indicated by the scale bar (nucleotide substitutions per site). All species-level branches have ultrafast bootstraps (UFBoot) support of 100. Green scores correspond to extended quadripartition internode certainty (EQP-IC) values. The clade containing all *Podospora* species other than *P. anserina* and *P. pauciseta* has an EQP-IC of 0.09, a value much lower compared to the analysis without an outgroup, likely as a result of instability in the position of *C. samala* across gene phylogenies.

Supplementary Table 1. Metadata and genome assembly statistics of all the strains included in this study.
